# Interventions for the empowerment of older people and informal caregivers in transitional care decision-making: short report of a systematic review

**DOI:** 10.1186/s12877-023-03813-5

**Published:** 2023-02-28

**Authors:** Lotan Kraun, Kristel De Vliegher, Moriah Ellen, Theo van Achterberg

**Affiliations:** 1 Nursing Departement, Wit-Gele Kruis van Vlaanderen, Brussels, Belgium; 2 KU Leuven, Department of Public Health and Primary Care, Academic Centre for Nursing and Midwifery, University of Leuven, Leuven, Belgium; 3 Department of Health Policy and Management, Guilford Glazer Faculty of Business and Management and Faculty of Health Sciences, Ben-Gurion University of the Negev, Be’er Sheva, Israel; 4 Institute of Health Policy Management and Evaluation, Dalla Lana School Of Public Health, University of Toronto, Toronto, Canada

**Keywords:** Aged, Decision-making, Empowerment, Transitional care

## Abstract

**Background:**

Care transitions across different settings necessitate careful decision-making for all parties involved, yet research indicates that older people and informal caregivers do not have a strong voice in such decisions.

**Objective:**

To provide a systematic overview of the literature about interventions designed to empower older people and informal caregivers in transitional care decision-making.

**Design:**

A systematic review (Prospero Protocol CRD42020167961; funded by the EU’s Horizon 2020 program).

**Data sources:**

Five databases were searched: PubMed, EMBASE, Web of Science, PsycINFO, and CINAHL.

**Review methods:**

The review included evaluations of empowerment in decision-making interventions for older people and informal caregivers facing care transitions, that were published from the inception of the databases up until April 2022. Data extractions were performed by two independent researchers and the quality of studies was assessed with the relevant JBI-critical appraisal tools. A narrative descriptive analysis of the results was performed.

**Findings:**

Ten studies, reporting on nine interventions, and including a total of 4642 participants, were included. Interventions included transition preparation tools, support from transition coaches, shared decision-making interventions, and advance care planning. Designs and outcomes assessed were highly diverse and showed a mix of positive and lacking effects.

**Conclusions:**

There is a lack of research on how to empower older people and their informal caregivers in transitional care decision-making. Empowerment in decision-making is usually not central in transitional care interventions, and effects on actual empowerment are mostly not assessed. Conclusions on how to empower older people and informal caregivers in transitional care decision-making cannot be drawn.

**Supplementary Information:**

The online version contains supplementary material available at 10.1186/s12877-023-03813-5.

## Background

Older people are at risk of low quality of life due to (comorbid) health conditions that can come with advanced age [1, 2]. As a result, care for complex health conditions across care settings, and transitions between these settings are often needed [3]. However, transitional care is often poorly handled [4, 5] and can lead to negative outcomes, low care satisfaction, and care inefficacy [6, 7]. Though older people should be central in such transitions, they report confusion, a lack of control, and the inability to have their say in the care transition decisions [8–10]. At the same time, informal caregivers report unsatisfactory communication with the older person they care for, within their families, as well as with health professionals, all leading to hindered transitional care decision-making [8]. A focus on the empowerment of older people and informal caregivers in the transitional care decision-making thus becomes relevant [11, 12].

The World Health Organization defines empowerment as “*a process through which people gain greater control over decisions and actions affecting their health*” [13]. In line with this definition, alternative empowerment interventions can be considered [14–16]. However, an overview of interventions for empowering older people and informal caregivers in transitional care decision-making, and their effects, is not available from the literature.

*Thus, we aim to provide a systematic overview of the literature concerning the evaluation of interventions designed to empower older people and informal caregivers in transitional care decision-making, and to explore their impact.*

## Methods

We performed a systematic review (Prospero Protocol CRD42020167961), and report its results in line with the PRISMA [17] guidelines.

### Review methods

PubMed, EMBASE, Web of Science, PsycINFO, and CINAHL were searched from the inception of the databases up until April 2022. Concepts for the search strategy were ‘old age’, ‘informal caregivers’, ‘involvement in decision-making’, ‘transitional care’, and ‘home’ as a location for either the start or the end of the transition. The search strategy was developed by all authors and search strings were built, pre-tested, and finalized with the help of a professional information specialist (see supplementary file 1).

During the process of searching and including literature we were in contact with various authors on the topic of transitional care (e.g. to obtain full text or additional info). Potentially relevant publications suggested by these authors were also checked for their relevance.

### Inclusion and exclusion criteria

Publications were included if they met the following criteria: (1) reports of empirical studies; (2) study participants (or at least 70% of them) aged 65+ and/or informal caregivers; (3) study participants facing a care transition departing from or returning to the older person’s home; and (4) reports should evaluate interventions that include empowerment in transitional care decision-making. Studies without empirical data were excluded. Language was not a reason for exclusion.

### Study selection

The first author (LK) performed the searches and removed duplicates. The selection process was always performed by two independent researchers per publication, first based on titles and abstracts and then based on full text screening for the remaining articles. In case of disagreements, the researchers tried to reach a consensus or consulted a third researcher where necessary.

### Quality assessment

Study quality was double blindly evaluated by two independent reviewers, using the relevant JBI-critical appraisal tools [18].

### Data extraction

Data extraction was independently conducted by LK and TvA, and for publication year, country, interventions for empowerment in decision-making, design, sample, outcomes measured, and main results (Table 1). References to details on the study interventions were always checked in the process. Discrepancies in the extractions were discussed and resolved.

**Table 1:** Study characteristics (*n* = 10), interventions, outcomes assessed and main results

Author year, country	Empowerment interventions	Design	Sample (age)	Outcomes	Main results
Adekpedjou, 2020, Canada [19]	*Shared decision-making on housing arrangements through caregiver training and supportive decision guide*	Cluster Randomized Trial	Informal caregivers of cognitively impaired older people Intv *n* = 138 (median = 60) Contr *n* = 158 (median = 63)	*Primary:* Proportion of caregivers self-reporting an active role in decision-making. *Secondary*: Preferred health-related housing option, decision made, decisional conflict, decision regret, and burden of care as perceived by caregivers.	• Non-significant intervention effect on self-reported active informal caregiver role in decision-making (79.6% in intv group vs 68.4% in contr group; 95% CI for 12% difference between groups − 2 to 27%; *P* = 0.10). • No difference between the intervention group and control for preferred option (% stay home) (50.7% vs 51.9%; *p* = 0.95). No difference between the intervention group and control for actual decision (% stay home) (19.6% vs 16.4%; *p* = 0.60). • No difference between the intervention group and control for decision regret (52.9% vs 48.7%; *p* = 0.60). • No difference between the intervention group and control for match between preferred option and actual decision (64.5% vs 58.2%; *p* = 0.28). • No difference between intervention group and control for decisional conflict and caregivers’ burden (mean of 29.10 vs 30.48; *p* = 0.62).
Coleman 2006, USA [20]	*Hospital discharge preparation tool + support of a transition coach*	Randomized controlled trial	Older people facing hospital discharge Intv *n* = 379 (mean = 76) Contr *n* = 371 (mean = 76)	*Primary:* Unplanned re-hospitalizations within 30, 90 and 180 days *Secondary:* Unplanned re-hospitalizations at 30, 60, and 90 days post-discharge for the same condition	• Fewer re-hospitalizations in the intervention group as compared to control within 30 days (8.3% vs 11.9%; OR = 0.59; 95% CI 0.35–1.00) and 90 days (16.7% vs 22.5%; OR = 0.64; 95% CI 0.42–0.99); no difference at 180 days (25.6% vs 30.7%; OR = 0.80; 95% CI 0.54–1.19). • Fewer re-hospitalizations for the same condition in the intervention group as compared to control within 90 days (5.3% vs 9.8%; OR = 0.50; 95% CI 0.26–0.96) and 180 days (8.6% vs 13.9%; OR = 0.55; 95% CI 0.30–0.99); no significant difference between study groups at 30 days (2.8% vs 4.6%; OR = 0.56 95% CI 0.24–1.31).
Coleman, 2004**,** USA [21]	*Hospital discharge preparation tool + support of a transition coach*	Non-randomized controlled trial	Older people facing hospital discharge Intv *n* = 158 (mean = 75) Contr *n* = 1235 (mean = 78)	*Primary:* Complicated care episodes (=transitions to more intense level of care) within 30 days post-discharge *Secondary:* Eight measures for re-hospitalization / ED admission	• No difference between the intervention group and control for complicated care episodes (9.5% vs 14.9%; OR = 0.74; 95% CI 0.38–1.46). • Fewer re-hospitalizations within 30, 60 and 90 days in the intervention group as compared to control (8.9% vs 13.8%; OR = 0.52; 95% CI 0.28–0.96; 13.5% vs 22.9%; OR = 0.43; 95% CI 0.25–0.72; and 22.9% vs 32.0%; OR = 0.57; 95% CI 0.36–0.92 respectively). • Fewer ED re-admissions within 90 days in the intervention group as compared to control (18.3% vs 25.7%; OR = 0.61; 95% CI 0.39–0.95). • No difference in ED-readmissions between the intervention group and control at 30 and 180 days (11.0% vs 14.2%; OR = 0.76; 95% CI 0.44–1.30; and 37.1% vs 36.0%; OR = 1.16; 95% CI 0.78–1.72 respectively).
Grimmer, 2006, Australia [22]	*Hospital discharge preparation tool*	Before-after study	Older people facing hospital discharge Intv *n* = 107 (mean = 70) Contr *n* = 210 (mean = 69)	*Primary:* Quality of preparation for discharged (self-assessed). *Secondary:* Experiences with managing at home, if hospital could have done more, and use of the tool.	• No significant differences in quality of discharge preparation (OR = 1.6 CI 95% 0.8–3.2) between pre- and post-cohorts; subjects in both cohorts were generally unaware of discharge plans made by hospital staff. • In the intervention group, 89% found the tool relevant. It empowered them to plan ahead and deal with practical issues of returning home.
Polt, 2019, Austria [23]	*Advance care planning for preferred place of death*	Retrospective comparative study	Older people with palliative care at (nursing) home Intv *n* = 38 (mean = 80) Contr 1 *n* = 65 (mea*n* = 74) Contr 2 *n* = 755 (mean = 74)	*Primary*: Place of death Secondary: Correspondence between preferred place of death and actual place of death	• Subjects in the intervention group more often died at home as compared to the control groups (72% vs. 53 and 57% for the two control groups; CIs or other statistical evaluations not given) • Preferred place of death strongly correlated with actual place of death (*p* = 0.02 - correlation not reported).
Preen 2005, Australia [24]	*Shared decision-making on a transition plan*	Randomized controlled trial	Older people facing hospital discharge Intv *n* = 91 (mean = 75) Contr *n* = 98 (mean = 75)	*Primary:* Quality of life at 7 days post-discharge *Secondary:* Fourteen aspects of satisfaction with transition at 7 days post-discharge	• No differences in physical quality of life between pre and post discharge in both intervention and control groups (− 2.2%, *P* = 0.45, − 3.9%, *p* = 0.28 respectively). • Significant improvement in mental quality of life in the intervention group (13.4%, *p* < 0.01). • No differences in mental quality of life between pre and post discharge in the control group 2.8%, *p* = 0.32) Improved of life aspects in intervention group. • Intervention subjects were more positive on their input in the discharge process (difference of 11.1%, *p* = 0.09) and achievability of care-arrangements (difference of 10.1%, *p* < 0.01), they more often thought discharge was better than earlier discharges (difference of 22.8%, *p* < 0.01).
Schusselé Filliettaz, 2021, Switzerland [25]	*Shared decision-making on a transition plan*	Observational, descriptive study	Older people with and without complex needs *n* = 453 (mean = 82)	*Fidelity*: Occurrence of 1) multilateral coordination processes, and 2) inter-professional and inter-institutional coordination meetings *Coverage*: Comparison of the processes and meeting for people with and without complex needs; involvement in the processes and meetings.	• Fidelity: multilateral coordination processes occurred in 65% and inter-professional and inter-institutional coordination meetings occurred in 15% of the cases. • Coverage: multilateral coordination processes occurred in 78% of people with complex needs vs 44% of people without complex needs (Chi2 = 57.09, *p* < 0.01); inter-professional and inter-institutional coordination meetings occurred in 24% of people with complex needs vs 1% of people without complex needs (Chi2 = 32.89; *p* < 0.01). • Older people and informal caregivers were involved in 82% of the inter-professional and inter-institutional coordination meetings and 24% of the inter-professional and inter-institutional coordination meetings.
Toles, 2017, USA [26]	*Shared decision-making on a transition plan*	A non-randomized, historically controlled design	Older people facing discharge form skilled nursing facility to home and their informal caregivers Intv *n* = 71 dyads Older people (mean 80) Informal caregivers (mean 64) Contr *n* = 74 dyads Older people (mean 80) Informal caregivers (mean 64)	*Primary:* Older persons’ and informal caregivers’ preparedness for discharge, assessed with Care-Transitions Measure-15. (CTM-15). *Secondary*: Feasibility based on chart reviews and relevance assessed with a survey for staff experiences.	• Intervention dyads, were more prepared for discharge as compared to dyads receiving usual care (CTM-15 score 74.7 vs 65.3, mean ratio 1.16, CI 95%: 1.08–1.24). • The intervention was feasible and relevant to skilled nursing facilities staff (i.e., 96.9% of staff recommended intervention use in the future).
Tsui, 2015, Canada [27]	*Hospital discharge preparation tool*	Observational, descriptive study	Community-dwelling older people, after hospitalization for hip fracture *n* = 31 (mean = 78) Informal carer *n* = 1 (NR)	*Primary:* Perceived utility of the discharge preparation tool. *Secondary*: Open-ended questions on the tool’s structure, organization, content, and illustrations used.	• High scores for utility (median of 9 on 10-point scale). • Medians > 4 on 5-point scale for all aspects of the tool. • Favorable comments on tool overall; some suggestions for additional content and how hospital staff could go over the items with them.
Ulin, 2016, Sweden [28]	*Shared decision-making on a transition plan*	Non-randomized controlled trial	Older people with worsening chronic heart failure facing hospital discharge Intv *n* = 125 (mean = 77) Contr *n* = 123 (mean = 80)	*Primary*: Days from hospital admission to 1st notice to community care services. Secondary: Days from admission to a) discharge planning conference; b) notification of discharge readiness.	• Fewer days from hospital admission to 1st notice to community care services in the intervention group, compared to control (2 days vs 14 days, *p* = NS). • Fewer days from admission to discharge planning conference in the intervention group, compared with the usual care (23 days vs 28 days, *p* = 0.03). • Fewer days from admission to notification of discharge readiness in the intervention group, compared with control (30 days vs 35 days, *p* = 0.01).

*NS* Not significant, *NR* Not reported, *OR* Odds ratio, *CI* Confidence interval

### Analysis and reporting

Given the limited number of studies and the considerable heterogeneity, a narrative descriptive analysis of the studies was performed and a short report was drawn-up.

## Findings

**Fig. 1:**
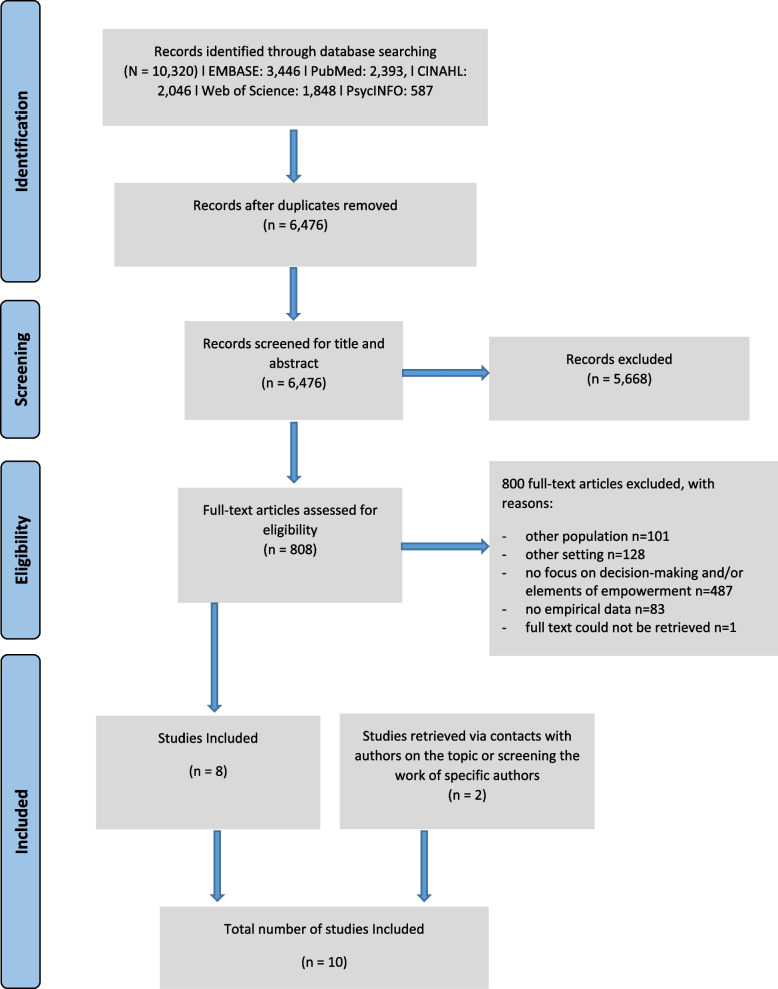
PRISMA flow diagram for the identification, selection, and inclusion of studies

Of 6476 unique records, full texts of 808 studies were screened. Eight of these were included. Two additional studies were retrieved through contacts with authors on the topic or screening the work of specific authors, resulting in a total of ten studies (total of 4642 participants) reporting on nine interventions (Fig. 1). The studies were three (cluster-) randomized controlled trials (RCTs), three non-RCTs, one retrospective comparative study, one before-after study, and two observational studies (Table 1).

### Quality assessment

No studies were excluded based on quality. Overall, studies were of a reasonable quality in the light of the designs used. However, for most studies one or a few study aspects were unclear from the report, or received a negative score. (See supplementary file 2 for details).

### The studies

Five out of the nine interventions addressed hospital discharge (Table 1). The other interventions focused on transitions from a short stay unit to outpatient/home care, people’s preferences for potential transfers in case of severely deteriorating health, and future housing decisions (i.e. living at home or in a residential care facility). The two latter studies were the only ones in which empowerment for decision-making was the central intervention. Empowerment was an element in a larger transitional care intervention in all other cases, where healthcare professionals were central in initiating and planning for transitions.

Intervention (elements) for empowerment included tools for considering and preparing for transitions, support from transition coaches, shared decision-making (SDM), and advanced care planning. Outcomes focused on intervention feasibility, use of care services, timeliness of arrangements, utility of the interventions, transition preparedness, and preferred place of death (Table 1).

### Interventions and effects

Hospital discharge preparation tools were operationalized as planning manuals and checklists that encourage people to consider all aspects of hospital discharge, necessary arrangements, and their personal discharge readiness. The two studies evaluating such tools as a single intervention showed peoples’ appreciation for the tools with a view to their relevance and utility, but indicated no effects on the quality of discharge [22, 27].

A combined intervention of a discharge preparation tool and support of a transition coach was evaluated in two studies [20, 21]. In these studies, the transition coach offered guidance and continuity of care at several points in the transition process. Results showed reduced use of emergency department services and fewer re-hospitalizations, but not consistently for all comparisons.

Shared decision-making interventions were central in five studies [19, 24–26, 28]. Four studies evaluated SDM on transition plans and included identifying problems and solutions, person-centered mutual goals development, and ongoing evaluation and follow-up [24, 25, 28]. Results included shorter hospital stays, fewer discharge delays, improved mental (but not physical) quality of life, and positive views on the older people’s involvement in discharge processes. Feasibility results from one of these studies indicated that coordination processes and actual involvement did not always happen. In one of the studies, inter-professional SDM training and use of a decision guide, were the core intervention elements [19]. This study reported a higher proportion of informal caregivers reporting an active role in the decision-making as compared to control, but not statistically significantly so, and no effects on secondary outcomes were found.

Advance care planning for preferred place of death [23], was a very brief intervention that asked people in palliative care to document their preferred place of death. In this retrospective comparative study, the intervention was associated with dying at home more often (as compared to people with no advanced care planning), and a positive correlation between preferred place of death and actual place of death was found. However, statistics for these results were incomplete in the study report.

## Discussion

Our review identified limited research on interventions for the empowerment of older people and informal caregivers at the time of transitional care decision-making. Shared decision-making, advanced care planning, and (combined) hospital and skilled nursing facilities discharge preparation tools and support from a transition coach have all been used for such empowerment. However, variability in interventions, study designs and outcomes assessed, and inconclusive results do not allow for drawing conclusions on their effectiveness.

Two interventions primarily focused on empowerment in decision-making and assessed relevant outcomes for empowerment [19, 23], while all of other interventions included elements of empowerment in decision-making in a larger multi-component intervention. This was also reflected by some of the primary outcomes for the intervention evaluation (e.g., looking at re-hospitalizations and emergency department visits, rather than person-centered outcomes). Such variability of outcomes assessed for the empowerment of older people was also reported by Shearer et al. [29]. Their review on empowerment of older people in taking health-related decisions, showed that outcomes assessed were highly variable, even when empowerment was conceptualized in the same way [29]. These and other findings illustrate that there is no generally accepted measurement of people’s empowerment [30], even though there is a clear need for a stronger emphasis on person-centered empowerment [14, 29].

This review’s strength lies in its exhaustive literature searches and rigorous inclusion and data extraction processes. However, a major limitation is that we could not synthesize findings, due to the high variability in interventions, designs used and outcomes assessed. Instead, we categorized the interventions into logical groups, and highlighted the different interventions and their outcomes.

In conclusion, this brief report indicates a lack of research on how to empower older people and their informal caregivers in transitional care decision-making. Furthermore, empowerment for decision-making is insufficiently central to transitional care interventions and effects on actual empowerment are mostly not assessed. As a result, conclusions on how best to empower older people and informal caregivers in transitional care decision-making cannot be drawn.

## Supplementary Information

**Additional file 1.** Search strategy and data sources.

**Additional file 2.** Quality assessment of the included studies using the JBI appraisal instruments.

## Data Availability

The datasets used and/or analysed during the current study available from the corresponding author on reasonable request.

## Abbreviations

EU: European Union
RCT: Randomized Controlled Trial
SDM: Shared Decision-Making
ED: Emergency Department
NS: Not significant
NR: Not reported
OR: Odds ratio
CI: Confidence interval
