# Supplementary material for: Interventions for the empowerment of older people and informal caregivers in transitional care decision-making: short report of a systematic review
Source: BMC Geriatr. 2023 Feb 28;23:113. doi: 10.1186/s12877-023-03813-5 (PMC9976408; doi:10.1186/s12877-023-03813-5)
Supplement: Supplementary file 1 — Additional file 1. Search strategy and data sources. [file 12877_2023_3813_MOESM1_ESM.docx]

**Supplementary 1 - Search strategy and data sources**

Search strategy and data sources

Five concepts were used to build the search strategy and to capture relevant scientific papers: ‘old age’, ‘informal caregivers’, ‘involvement in decision-making’, ‘transitional care’, and ‘home as a location for the start or the end of the transition’. Five databases (PubMed, Embase, CINHAL, Web of Science, and PsycInfo) were systematically searched for literature that was published from the inception of the databases up to April 2022. The search string was created by combining Index Terms (e.g., MeSH for PubMed, such as “aged”, “caregivers”, “patient participation”, and “transitional care”) and “natural” terms (e.g., “elderly”, “carer”, “patient involvement”, “decision”, and “transition of care”).

1. **PubMed (2,393)**

("Aged"[MeSH] OR Aged[tiab] OR “Aged, 80 and over”[tiab] OR Elderly[tiab] OR elders[tiab] OR Senior-citizen*[tiab] OR Advanced-age[tiab] OR Old [tiab] OR older[tiab] OR geriatric*[tiab] OR octogenarian*[tiab] OR nonagenarian*[tiab] OR centenarian*[tiab] OR septuagenarian*[tiab])

**OR**

("caregivers"[MeSH] OR "spouses"[MeSH] OR "family"[MeSH] OR "friends"[MeSH] OR “geriatric nursing”[MeSH] OR caregiver*[tiab] OR (care[tiab] AND giver*[tiab]) OR spouse*[tiab] OR spousal[tiab] OR family[tiab] OR families[tiab] OR friends[tiab] OR friend[tiab] OR "geriatric nursing"[tiab] OR informal-care*[tiab] OR partner*[tiab] OR wife[tiab] OR wifes[tiab] OR wives[tiab] OR carer[tiab] OR carers[tiab])

**AND**

(“patient participation”[MeSH] OR “Decision making”[MeSH] OR "Patient-Centered Care"[MeSH] OR “Patient preference”[MeSH] OR “patient participation”[tiab] OR “patients participation”[tiab] OR decision-making*[tiab] OR “patient-centered care”[tiab] OR (patients[tiab] AND "centered care"[tiab]) OR “patient-centred care”[tiab] OR patient-preference*[tiab] OR patients-preference*[tiab] OR “Patient empowerment”[tiab] OR “Patients empowerment”[tiab] OR “Consumer participation”[tiab] OR “Patient activation”[tiab] OR “Patient engagement”[tiab] OR “Patients engagement”[tiab] OR “Shared decision making”[tiab] OR “Patient involvement”[tiab] OR “Patients involvement”[tiab] OR Patient-perspective*[tiab] OR Patients-perspective*[tiab] OR “Patient's perspective”[tiab] OR ((patient[tiab] OR patients[tiab]) AND decision*[tiab]) OR “Consumer perspective”[tiab] OR “Consumers perspective”[tiab] OR “Consumer's perspective”[tiab] OR Patient-view* [tiab] OR “Patient’s view”[tiab] OR Patients-view*[tiab] OR “Patient's expression”[tiab] OR “Consumer involvement”[tiab] OR (consumers[tiab] AND involvement[tiab]) OR “patient’s needs”[tiab] OR “patient need”[tiab] OR “patients need”[tiab] OR “Patients needs”[tiab] OR “Patient needs”[tiab] OR “patient’s need”[tiab])

**AND**

("Transitional Care"[Mesh] OR "patient discharge"[Mesh] OR “patient admission”[MeSH] OR "transitional care"[tiab] OR “transition of care”[tiab] OR patient-admission*[tiab] OR patient-discharge*[tiab] OR "continuity of care”[tiab] OR "care continuity"[tiab] OR care-transition*[tiab] OR "coordination of care”[tiab] OR "hospital to home”[tiab] OR "home to hospital”[tiab] OR admission[tiab] OR admissions[tiab] OR transfer[tiab] OR transfers[tiab] OR “home discharge”[tiab] OR “discharge planning”[tiab] OR patient-admittance*[tiab])

**AND**

(“Home Care Services”[Mesh] OR "Adult Day Care Centers"[Mesh] OR "Primary Health Care"[Mesh:NoExp] OR “senior center”[Mesh] OR "senior center"[tiab]) OR “community"[tiab] OR "in home"[tiab] OR "from home"[tiab] OR "home based"[tiab] OR "at home”[tiab] OR “home care”[tiab] OR “home discharge”[tiab] OR “day-care”[tiab] OR daycare[tiab] OR Home-service*[tiab] OR residential[tiab] OR primary-care[tiab])

1. **EMBASE (3,446)**

‘Aged‘/exp OR ‘elderly’:ti,ab,kw OR ‘senior citizen*’:ti,ab,kw OR ‘80 and over’:ti,ab,kw OR ‘centenarian*’:ti,ab,kw OR ‘nonagenerian*’:ti,ab,kw OR ‘octogenarian*’:ti,ab,kw OR ‘advanced age’:ti,ab,kw OR ‘old’:ti,ab,kw OR ‘septuagenarian*’:ti,ab,kw OR ‘sexagenarian*’:ti,ab,kw OR aged:ti,ab,kw OR ‘older’:ti,ab,kw OR geriatric*:ti,ab,kw

**OR**

‘caregiver’/exp OR ‘geriatric nursing’/exp OR ‘caregiver support’/exp OR ‘family’/exp OR ‘family centered care’/exp OR ‘friend’/exp OR ‘care giver’:ti,ab,kw OR ‘caregiver’:ti,ab,kw OR ‘carer’:ti,ab,kw OR ‘carers’:ti,ab,kw OR ‘informal care*’:ti,ab,kw OR ‘family’:ti,ab,kw OR ‘families’:ti,ab,kw OR ‘spouse*’:ti,ab,kw OR ‘spousal’:ti,ab,kw OR ‘wife’:ti,ab,kw OR wifes:ti,ab,kw OR wives:ti,ab,kw OR ‘partner*’:ti,ab,kw OR ‘friend’:ti,ab,kw OR ‘friends’:ti,ab,kw

**AND**

‘patient decision making’/exp OR ‘patient participation’/exp OR ‘decision making’/exp OR ‘patient preference’/exp OR ‘patient participation’:ti,ab,kw OR ‘patients participation’:ti,ab,kw OR ‘decision-making*’:ti,ab,kw OR ‘patient centered care’:ti,ab,kw OR ‘patients centered care’:ti,ab,kw OR ‘patient centred care’:ti,ab,kw OR ‘patient* preference*’:ti,ab,kw OR ‘Patient* empowerment’:ti,ab,kw OR ‘Consumer participation’:ti,ab,kw OR ‘Patient activation’:ti,ab,kw OR ‘Patient* engagement’:ti,ab,kw OR ‘Shared decision making’:ti,ab,kw OR ‘Patient* involvement’:ti,ab,kw OR ‘Patient* perspective’:ti,ab,kw OR 'patient s perspective':ti,ab,kw OR ((‘patient’:ti,ab,kw OR ‘patients’:ti,ab,kw AND ‘decision*’:ti,ab,kw) OR ‘Consumer* perspective’:ti,ab,kw OR ‘Consumers perspective’:ti,ab,kw OR ‘Patient view’:ti,ab,kw OR ‘Patient* view’:ti,ab,kw OR ‘Patient* expression’:ti,ab,kw OR ‘Consumer involvement’:ti,ab,kw) OR ‘consumers involvement’:ti,ab,kw OR ‘patient* needs’:ti,ab,kw

**AND**

‘hospital admission’/exp OR ‘hospital discharge’/exp OR ‘transitional care’/exp OR ‘discharge planning’:ti,ab,kw OR ‘patient discharge*’:ti,ab,kw OR ‘patients discharge’:ti,ab,kw OR ‘patient admittance*’:ti,ab,kw OR ‘admission*’:ti,ab,kw OR ‘transitional care’:ti,ab,kw OR ‘transition care’:ti,ab,kw OR ‘care transition*’:ti,ab,kw OR ‘transition of care’:ti,ab,kw OR ‘continuity of care’:ti,ab,kw OR ‘coordination of care’:ti,ab,kw OR ‘hospital to home’:ti,ab,kw OR 'home to hospital':ti,ab,kw OR ‘home discharge’:ti,ab,kw OR ‘care continuity’:ti,ab,kw

**AND**

‘Home care’/exp OR ‘senior center’/exp OR ‘adult day care’/exp OR 'primary health care'/exp OR 'in home':ti,ab,kw OR 'home based':ti,ab,kw OR 'at home':ti,ab,kw OR 'home care':ti,ab,kw OR 'day care':ti,ab,kw OR ‘daycare’:ti,ab,kw OR 'Home service*':ti,ab,kw OR ‘residential’:ti,ab,kw OR ‘community’:ti,ab,kw OR ‘primary care’:ti,ab,kw OR ‘senior center’:ti,ab,kw OR ‘from home’:ti,ab,kw

1. **Web of Science (1,848)**

(Aged OR “Aged, 80 and over” OR Elderly OR elders OR Senior-citizen* OR Advanced-age OR Old OR older OR geriatric* OR octogenarian* OR nonagenarian* OR centenarian* OR septuagenarian*)

**OR**

(Friend OR Friends OR “geriatric nursing” OR Caregiver OR caregivers OR “Care giver” OR “care givers” OR “informal caregiver” OR “informal caregivers” OR “family caregiver” OR “family caregivers” OR informal-care* OR “family carer” OR “family carers” OR “Informal-care” OR Spouse* OR Spousal OR Family OR Families OR Husband* OR Partner* OR Wife*)

**AND**

(“patient participation” OR “patients participation” OR decision-making* OR “patient-centered care” OR (patients AND "centered care") OR “patient-centred care” OR “patient preference” OR “patient preferences” OR “patients preference” OR “patients preferences” OR “Patient empowerment” OR “Patients empowerment” OR “Consumer participation” OR “Patient activation” OR “Patient engagement” OR “Patients engagement” OR “Shared decision making” OR “Patient involvement” OR “Patients involvement” OR Patient-perspective* OR Patients-perspective* OR Patient's-perspective* OR ((patient OR patients) AND decision*) OR “Consumer perspective” OR “Consumers perspective” OR “Consumer's perspective” OR “Patient view” OR “Patient’s view” OR “Patients view” OR “Patient's expression” OR “Consumer involvement” OR (consumers AND involvement) OR “patient’s needs” OR “patient need” OR “patients need” OR “Patients needs” OR “Patient needs” OR “patient’s need”)

**AND**

("transitional care" OR “transition of care” OR patient-admission* OR patient-discharge* OR "continuity of care” OR "care continuity" OR care-transition* OR "coordination of care” OR "hospital to home” OR "home to hospital” OR admission OR admissions OR transfer OR transfers OR “home discharge” OR “discharge planning” OR patient-admittance*)

**AND**

(“senior center” OR "community" OR "in home" OR "from home" OR "home based" OR "at home” OR “home care” OR “home discharge” OR “day-care” OR daycare OR Home-service* OR residential OR primary-care)

1. **PsycINFO (587)**

((DE "Geriatric Patients") OR (DE "Geriatrics") OR (DE “gerontology”) OR (TI (Aged OR “Aged, 80 and over” OR Elderly OR elders OR Senior-citizen* OR Advanced-age OR Old OR older OR geriatric* OR octogenarian* OR nonagenarian* OR centenarian* OR septuagenarian*) OR (AB (Aged OR “Aged, 80 and over” OR Elderly OR elders OR Senior-citizen* OR Advanced-age OR Old OR older OR geriatric* OR octogenarian* OR nonagenarian* OR centenarian* OR septuagenarian*))

**OR**

((DE "caregivers") OR (DE "spouses") OR (DE "family") OR (TI (Friend OR Friends OR “geriatric nursing” OR Caregiver OR caregivers OR “Care giver” OR “care givers” OR “informal caregiver” OR “informal caregivers” OR “family caregiver” OR “family caregivers” OR informal-care* OR “family carer” OR “family carers” OR “Informal-care” OR Spouse* OR Spousal OR Family OR Families OR Husband* OR Partner* OR Wife*) OR (AB (Friend OR Friends OR “geriatric nursing” OR Caregiver OR caregivers OR “Care giver” OR “care givers” OR “informal caregiver” OR “informal caregivers” OR “family caregiver” OR “family caregivers” OR informal-care* OR “family carer” OR “family carers” OR Informal-care OR Spouse* OR Spousal OR Family OR Families OR Husband* OR Partner* OR Wife*))

**AND**

((DE (“Patient Participation”) OR (DE “Decision making”) OR (TI (“patient participation” OR “patients participation” OR decision-making* OR “patient-centered care” OR (patients AND "centered care") OR “patient-centred care” OR “patient preference” OR “patient preferences” OR “patients preference” OR “patients preferences” OR “Patient empowerment” OR “Patients empowerment” OR “Consumer participation” OR “Patient activation” OR “Patient engagement” OR “Patients engagement” OR “Shared decision making” OR “Patient involvement” OR “Patients involvement” OR Patient-perspective* OR Patients-perspective* OR Patient’s-perspective* OR ((patient OR patients) AND decision*) OR “Consumer perspective” OR “Consumers perspective” OR “Consumer's perspective” OR “Patient view” OR “Patient’s view” OR “Patients view” OR “Patient's expression” OR “Consumer involvement” OR (consumers AND involvement) OR “patient’s needs” OR “patient need” OR “patients need” OR “Patients needs” OR “Patient needs” OR “patient’s need”) OR (AB (“patient participation” OR “patients participation” OR decision-making* OR “patient-centered care” OR (patients AND "centered care") OR “patient-centred care” OR “patient preference” OR “patient preferences” OR “patients preference” OR “patients preferences” OR “Patient empowerment” OR “Patients empowerment” OR “Consumer participation” OR “Patient activation” OR “Patient engagement” OR “Patients engagement” OR “Shared decision making” OR “Patient involvement” OR “Patients involvement” OR “Patient perspective” OR “Patients perspective” OR “Patient's perspective” OR ((patient OR patients) AND decision*) OR “Consumer perspective” OR “Consumers perspective” OR “Consumer's perspective” OR “Patient view” OR “Patient’s view” OR “Patients view” OR “Patient's expression” OR “Consumer involvement” OR (consumers AND involvement) OR “patient’s needs” OR “patient need” OR “patients need” OR “Patients needs” OR “Patient needs” OR “patient’s need”)

**AND**

((DE "Facility Admission") OR ("Discharge Planning") OR (DE "Client Transfer") OR (DE "Hospital Discharge") OR (DE "Continuum of Care") OR (TI ("transitional care" OR “transition of care” OR patient-admission* OR patient-discharge* OR "continuity of care” OR "care continuity" OR care-transition* OR "coordination of care” OR "hospital to home” OR "home to hospital” OR admission OR admissions OR transfer OR transfers OR “home discharge” OR “discharge planning” OR patient-admittance*) OR (AB ("transitional care" OR “transition of care” OR patient-admission* OR patient-discharge* OR "continuity of care” OR "care continuity" OR care-transition* OR "coordination of care” OR "hospital to home” OR "home to hospital” OR admission OR admissions OR transfer OR transfers OR “home discharge” OR “discharge planning” OR patient-admittance*)

**AND**

((DE "Adult Day Care") OR (DE "Home Care") OR (DE "Communities") OR (TI (“senior center” OR "community" OR "in home" OR "from home" OR "home based" OR "at home” OR “home care” OR “home discharge” OR “day-care” OR daycare OR Home-service* OR residential OR primary-care) OR (AB (“senior center” OR "community" OR "in home" OR "from home" OR "home based" OR "at home” OR “home care” OR “home discharge” OR “day-care” OR daycare OR Home-service* OR residential OR primary-care)

1. **CINAHL (2,046)**

(MH “Aged+”) OR (TI (Aged OR “Aged, 80 and over” OR Elderly OR elders OR Senior-citizen* OR Advanced-age OR Old OR older OR geriatric* OR octogenarian* OR nonagenarian* OR centenarian* OR septuagenarian*) OR (AB (Aged OR “Aged, 80 and over” OR Elderly OR elders OR Senior-citizen* OR Advanced-age OR Old OR older OR geriatric* OR octogenarian* OR nonagenarian* OR centenarian* OR septuagenarian*)

**OR**

((MH “Caregivers”) OR (MH “family”) OR (MH “spouses”) OR (TI (Friend OR Friends OR “geriatric nursing” OR Caregiver OR caregivers OR “Care giver” OR “care givers” OR “informal caregiver” OR “informal caregivers” OR “family caregiver” OR “family caregivers” OR informal-care* OR “family carer” OR “family carers” OR “Informal-care” OR Spouse* OR Spousal OR Family OR Families OR Husband* OR Partner* OR Wife*)) OR (AB (Friend OR Friends OR “geriatric nursing” OR Caregiver OR caregivers OR “Care giver” OR “care givers” OR “informal caregiver” OR “informal caregivers” OR “family caregiver” OR “family caregivers” OR informal-care* OR “family carer” OR “family carers” OR “Informal-care” OR Spouse* OR Spousal OR Family OR Families OR Husband* OR Partner* OR Wife*))

**AND**

((MH “Decision making“) OR (MH “Patient preference“) OR (MH “Consumer participation“) OR (MH “Decision making, shared“) OR (MH “patient centered care”) OR (MH “Community networks”) OR (TI (“patient participation” OR “patients participation” OR decision-making* OR “patient-centered care” OR (patients AND "centered care") OR “patient-centred care” OR “patient preference” OR “patient preferences” OR “patients preference” OR “patients preferences” OR “Patient empowerment” OR “Patients empowerment” OR “Consumer participation” OR “Patient activation” OR “Patient engagement” OR “Patients engagement” OR “Shared decision making” OR “Patient involvement” OR “Patients involvement” OR Patient-perspective* OR Patients-perspective* OR Patient's-perspective* OR ((patient OR patients) AND decision*) OR “Consumer perspective” OR “Consumers perspective” OR “Consumer's perspective” OR “Patient view” OR “Patient’s view” OR “Patients view” OR “Patient's expression” OR “Consumer involvement” OR (consumers AND involvement) OR “patient’s needs” OR “patient need” OR “patients need” OR “Patients needs” OR “Patient needs” OR “patient’s need”) OR (AB (“patient participation” OR “patients participation” OR decision-making* OR “patient-centered care” OR (patients AND "centered care") OR “patient-centred care” OR “patient preference” OR “patient preferences” OR “patients preference” OR “patients preferences” OR “Patient empowerment” OR “Patients empowerment” OR “Consumer participation” OR “Patient activation” OR “Patient engagement” OR “Patients engagement” OR “Shared decision making” OR “Patient involvement” OR “Patients involvement” OR “Patient perspective” OR “Patients perspective” OR “Patient's perspective” OR ((patient OR patients) AND decision*) OR “Consumer perspective” OR “Consumers perspective” OR “Consumer's perspective” OR “Patient view” OR “Patient’s view” OR “Patients view” OR “Patient's expression” OR “Consumer involvement” OR (consumers AND involvement) OR “patient’s needs” OR “patient need” OR “patients need” OR “Patients needs” OR “Patient needs” OR “patient’s need”)

**AND**

((MH “Transitional care“) OR (MH “Continuity of patient care+”) OR (MH “Patient discharge“) OR (MH “Tranfer, discharge“) OR (MH “Early Patient discharge”) OR (MH “Discharge planning“) OR (MH “transitional programs”) OR (MH “patient admission +”) OR (MH “patient discharge+”) OR (TI ("transitional care" OR “transition of care” OR patient-admission* OR patient-discharge* OR "continuity of care” OR "care continuity" OR care-transition* OR "coordination of care” OR "hospital to home” OR "home to hospital” OR admission OR admissions OR transfer OR transfers OR “home discharge” OR “discharge planning” OR patient-admittance*) OR (AB ("transitional care" OR “transition of care” OR patient-admission* OR patient-discharge* OR "continuity of care” OR "care continuity" OR care-transition* OR "coordination of care” OR "hospital to home” OR "home to hospital” OR admission OR admissions OR transfer OR transfers OR “home discharge” OR “discharge planning” OR patient-admittance*)

**AND**

((MH "Community Living") OR (MH "Day Care") OR (TI (“senior center” OR "community" OR "in home" OR "from home" OR "home based" OR "at home” OR “home care” OR “home discharge” OR “day-care” OR daycare OR Home-service* OR residential OR primary-care) OR (AB (“senior center” OR "community" OR "in home" OR "from home" OR "home based" OR "at home” OR “home care” OR “home discharge” OR “day-care” OR daycare OR Home-service* OR residential OR primary-care)
