# Supplementary material for: Interventions for the empowerment of older people and informal caregivers in transitional care decision-making: short report of a systematic review
Source: BMC Geriatr. 2023 Feb 28;23:113. doi: 10.1186/s12877-023-03813-5 (PMC9976408; doi:10.1186/s12877-023-03813-5)
Supplement: Supplementary file 2 — Additional file 2. Quality assessment of the included studies using the JBI appraisal instruments. [file 12877_2023_3813_MOESM2_ESM.docx]

**Supplementary 2 - Quality assessment of the included studies using the JBI appraisal instruments:**

| Design | Author | Critical appraisal questions* | | | | | | | | | | | | |
| --- | --- | --- | --- | --- | --- | --- | --- | --- | --- | --- | --- | --- | --- | --- |
|  |  | 1 | 2 | 3 | 4 | 5 | 6 | 7 | 8 | 9 | 10 | 11 | 12 | 13 |
| Randomized Controlled Trials | Adekpedjou et al. 2020 (1) | Y | U | Y | Y | N | N | U | Y | U | Y | Y | Y | Y |
|  | Coleman et al. 2006 (2) | Y | N | Y | U | Y | N | Y | Y | Y | Y | Y | Y | Y |
|  | Preen et al. 2005 (3) | Y | Y | Y | U | N | U | Y | Y | U | Y | Y | Y | Y |
| Quasi-experimental studies | Coleman et al. 2004 (4) | Y | U | Y | Y | NA | U | Y | Y | Y |  |  |  |  |
|  | Grimmer et al. 2006 (5) | Y | U | U | Y | NA | Y | Y | Y | Y |  |  |  |  |
|  | Ulin et al. 2016 (6) | Y | Y | U | Y | NA | Y | Y | Y | Y |  |  |  |  |
|  | Polt et al. 2019 (7) | Y | U | Y | Y | N | Y | Y | Y | U |  |  |  |  |
|  | Toles et al. 2017 (8) | Y | N | U | Y | Y | Y | Y | Y | Y |  |  |  |  |
| Analytical Cross Sectional Studies | Tsui et al. 2015 (9) | Y | Y | N | NA | NA | NA | Y | Y |  |  |  |  |  |
|  | Schusselé Filliettaz et al. 2021 (10) | Y | Y | Y | NA | NA | NA | Y | Y |  |  |  |  |  |

Y = yes; N = no; U = unclear; NA = not applicable

***JBI Critical Appraisal Checklist for Randomized Controlled Trials**

1 Was true randomization used for assignment of participants to treatment groups?

2 Was allocation to treatment groups concealed?

3 Were treatment groups similar at the baseline?

4 Were participants blind to treatment assignment?

5 Were those delivering treatment blind to treatment assignment?

6 Were outcomes assessors blind to treatment assignment?

7 Were treatment groups treated identically other than the intervention of interest?

8 Was follow-up complete and if not, were differences between groups in terms of their follow-up adequately described and analyzed?

9 Were participants analyzed in the groups to which they were randomized?

10 Were outcomes measured in the same way for treatment groups?

11 Were outcomes measured in a reliable way?

12 Was appropriate statistical analysis used?

13 Was the trial design appropriate, and any deviations from the standard RCT design (individual randomization, parallel groups) accounted for in the conduct and analysis of the trial?

***Quasi-experimental studies (non-randomized experimental studies)**

1 Is it clear in the study what is the ‘cause’ and what is the ‘effect’ (i.e. there is no confusion about which variable comes first)?

2 Were the participants included in any comparisons similar?

3 Were the participants included in any comparisons receiving similar treatment/care, other than the exposure or intervention of interest?

4 Was there a control group?

5 Were there multiple measurements of the outcome both pre and post the intervention/exposure?

6 Was follow-up complete and if not, were differences between groups in terms of their follow up adequately described and analyzed?

7 Were the outcomes of participants included in any comparisons measured in the same way?

8 Were outcomes measured in a reliable way?

9 Was appropriate statistical analysis used?

***JBI Checklist for Analytical Cross Sectional Studies**

1 Were the criteria for inclusion in the sample clearly defined?

2 Were the study subjects and the setting described in detail?

3 Was the exposure measured in a valid and reliable way?

4 Were objective, standard criteria used for measurement of the condition?

5 Were confounding factors identified?

6 Were strategies to deal with confounding factors stated?

7 Were the outcomes measured in a valid and reliable way?

8 Was appropriate statistical analysis used?

1. Adekpedjou R, Stacey D, Brière N, Freitas A, Garvelink MM, Dogba MJ, et al. Engaging Caregivers in Health-Related Housing Decisions for Older Adults With Cognitive Impairment: A Cluster Randomized Trial. Gerontologist. 2020;60(5):947-57.

2. Coleman EA, Parry C, Chalmers S, Min SJ. The care transitions intervention: results of a randomized controlled trial. Arch Intern Med. 2006;166(17):1822-8.

3. Preen DB, Bailey BE, Wright A, Kendall P, Phillips M, Hung J, et al. Effects of a multidisciplinary, post-discharge continuance of care intervention on quality of life, discharge satisfaction, and hospital length of stay: a randomized controlled trial. Int J Qual Health Care. 2005;17(1):43-51.

4. Coleman EA, Smith JD, Frank JC, Min SJ, Parry C, Kramer AM. Preparing patients and caregivers to participate in care delivered across settings: the Care Transitions Intervention. Journal of the American Geriatrics Society. 2004;52(11):1817-25.

5. Grimmer KA, Dryden LR, Puntumetakul R, Young AF, Guerin M, Deenadayalan Y, et al. Incorporating patient concerns into discharge plans: evaluation of a patient-generated checklist. Internet Journal of Allied Health Sciences and Practice. 2006;4(2):7.

6. Ulin K, Olsson LE, Wolf A, Ekman I. Person-centred care - An approach that improves the discharge process. Eur J Cardiovasc Nurs. 2016;15(3):e19-26.

7. Polt G, Weixler D, Bauer N. [A retrospective study about the influence of an emergency information form on the place of death of palliative care patients]. Wien Med Wochenschr. 2019;169(15-16):356-63.

8. Toles M, Colón-Emeric C, Naylor MD, Asafu-Adjei J, Hanson LC. Connect-Home: Transitional Care of Skilled Nursing Facility Patients and their Caregivers. J Am Geriatr Soc. 2017;65(10):2322-8.

9. Tsui K, Fleig L, Langford DP, Guy P, MacDonald V, Ashe MC. Exploring older adults' perceptions of a patient-centered education manual for hip fracture recovery: "everything in one place". Patient Prefer Adherence. 2015;9:1637-45.

10. Schusselé Filliettaz S, Moiroux S, Marchand G, Gilles I, Peytremann-Bridevaux I. Transitional shared decision-making processes for patients with complex needs: A feasibility study. J Eval Clin Pract. 2021;27(6):1326-34.
